# Serum biomarkers of delirium in the elderly: a narrative review

**DOI:** 10.1186/s13613-019-0548-1

**Published:** 2019-07-01

**Authors:** Katharina Toft, Janna Tontsch, Salim Abdelhamid, Luzius Steiner, Martin Siegemund, Alexa Hollinger

**Affiliations:** 1 Department for Anesthesia, Intensive Care and Emergency Medicine, See-Spital, Horgen, Kilchberg, Switzerland; 2 Institute for Anesthesia and Intensive Care, Hirslanden Klinik Zurich, Zurich, Switzerland; 3 Department for Intensive Care, University Hospital Basel, Basel, Switzerland; 4 Medical Faculty of the University of Basel, Basel, Switzerland; 5 Department for Anesthesia, Prehospital Emergency Medicine and Pain Therapy, University Hospital Basel, Basel, Switzerland

## Abstract

Delirium after surgery and in the intensive care unit (ICU) remains a challenge for patients, families, and caregivers. Over the years, many promising biomarkers have been investigated as potential instruments for risk stratification of delirium. This review aimed to identify and assess the clinical usefulness of candidate serum biomarkers associated with hospital delirium in patients aged 60 years and older. We performed a time-unlimited review of publications indexed in PubMed, Cochrane, Embase, and MEDLINE databases until June 2019 that evaluated baseline and/or longitudinal biomarker measurements in patients suffering from delirium at some point during their hospital stay. A total of 32 studies were included in this review reporting information on 7610 patients. Of these 32 studies, twenty-four studies reported data from surgical patients including four studies in ICU cohorts, five studies reported data from medical patients (1026 patients), and three studies reported data from a mixed cohort (1086 patients), including one study in an ICU cohort. Findings confirm restricted clinical usefulness to predict or diagnose delirium due to limited evidence on which biomarkers can be used and limited availability due to non-routine use.

**Electronic supplementary material:**

The online version of this article (10.1186/s13613-019-0548-1) contains supplementary material, which is available to authorized users.

## Introduction

Postoperative and intensive care unit (ICU) delirium remains a challenge for patients, families, and caregivers. Identified more than half a century ago in cardiac surgery patients, delirium today is characterized by criteria of the Diagnostic and Statistical Manual of Mental Disorders (DSM)-V, which can be summarized as a fluctuating disturbance of consciousness evolving over a short period of time, a change in cognition, and evidence from the current history, physical examination, or laboratory findings that the disturbance is caused by the direct physiological consequences of a general medical condition.

The suffering of delirious patients is severe as they may be restless, hallucinate, and be filled with fear. Unfortunately, problems continue even after the resolution of delirium. This syndrome may be associated with prolonged ICU and hospital stay [1, 2], more hospital readmissions [3], reduced quality of life, loss of independence, and increased mortality [1, 4–7]. Furthermore, the duration of delirium is associated with worse long-term cognitive function [8, 9]. The increased socioeconomic burden should also not be underestimated [10]. However, a recently updated delirium guidance paper on prevention and management of pain, agitation/sedation, delirium, immobility, and sleep disruption in adult ICU patients summarizes that delirium in critically ill adults has not been consistently shown to be associated with ICU length of stay, discharge disposition to a place other than home, depression, functionality/dependence, or mortality [11].

Ranging from 10 to 80% [12–14] or even up to 90% depending on the type of surgery [15], the overall incidence of delirium is high during hospital stay, especially in elderly patients. Delirium usually develops within 72 h after surgery and/or ICU admission. However, its impact is likely to be underestimated due to the predominance of hypoactive delirium.

Although the pathophysiology of delirium remains poorly understood, we know that the pathogenesis of the cognitive impairments associated with delirium is multifactorial. Certain entities such as drug overdose [16] but also drug withdrawal [17] bear delirium risk. As with so many disparate etiologies, it is highly unlikely that a single mechanism is solely responsible [18]. Therefore, research focuses on the assessment of modifiable pre-, intra-, and postoperative risk factors (e.g., dehydration, fluid balance, immobilization, analgesia, and sleep deprivation) associated with delirium, [18] as well as prediction, prevention, early detection, and treatment of this common psychiatric syndrome. One promising approach is the detection of elevated or lowered biomarkers as predictors or indicators of delirium [19, 20]. Furthermore, serum biomarkers may aid in risk stratification, diagnosis, and monitoring of delirium [19] and, finally, may help to find an effective treatment. This review aims to summarize the current state of knowledge on serum biomarkers of delirium.

## Methods

We performed an updated review on biomarkers of delirium based on previous publications [19]. As age is one of the most consistently reported risk factors for developing delirium, we restricted our search to publications including patients aged 60 years and older [18, 21]. Study selection and quality assessment were performed by two independent authors (AH and KT). The results were compared, and disagreements were reviewed (MS).

### Literature search

An electronic search of PubMed, Cochrane, Embase, and MEDLINE databases was performed. The detailed search strategy is available in “Appendix” (Additional files). Search terms used for each biomarker are listed in Additional file 1: Table S1. Every biomarker term according to Additional file 1: Table S1 was searched with “delirium”, “acute brain dysfunction”, “stroke”, “hemorrhagic stroke”, “ischemic stroke”, “traumatic brain injury”, and “septic encephalopathy”. The date of the last search was June 1, 2019.

### Inclusion and exclusion criteria

Only studies that met the following criteria were included: patients aged 60 or older, sample size of 10 or higher, use of standardized approach to diagnose delirium [e.g., Intensive Care Delirium Screening Checklist (ICDSC), Confusion Assessment Method (CAM or CAM-ICU); Nursing Delirium Screening Scale (NuDESC); Delirium Observation Screening Scale (DOS); Diagnostic and Statistical Manual of Mental Disorders (DSM)-III/IV; Delirium Symptom Interview (DSI); Delirium Rating Scale Revised-98-T (DRS-R98-T); Memorial Delirium Assessment Scale (MDAS)], ICU/hospital cohort (e.g., excluding studies performed in nursing homes), and English language. Reviews from all cohorts (i.e., medical, surgical, mixed, ICU) were included. Age limitation was chosen due to significantly higher reported incidence of delirium in patients 60 to 65 and older, and to help clarify results within the flood of information on delirium biomarkers available to date for the age category at highest risk. Studies reporting data that included patients with cognitive dysfunction due to preexisting psychiatric disorders, known dementia, or alcohol-related delirium (delirium tremens) were excluded. Reviews/meta-analyses, and studies reporting animal data or cerebrospinal fluid (CSF) biomarkers were also excluded.

### Data extraction

Study-relevant information was extracted by two independent investigators (AH and KT) for each included study. Any conflict of opinion was resolved by consensus with a third party (MS). Study location and date of study conduct, patient characteristics, past medical history including drug therapy prior to hospitalization, risk assessment scores (e.g., Charlson comorbidity index), outcome data (i.e., ICU and hospital length of stay, mortality), total number of patients, and study-specific procedures—including drug therapy during ICU and/or hospital stay—were considered relevant for data extraction. Observational and interventional study design was distinguished.

## Results

### Trial identification

**Fig. 1:**
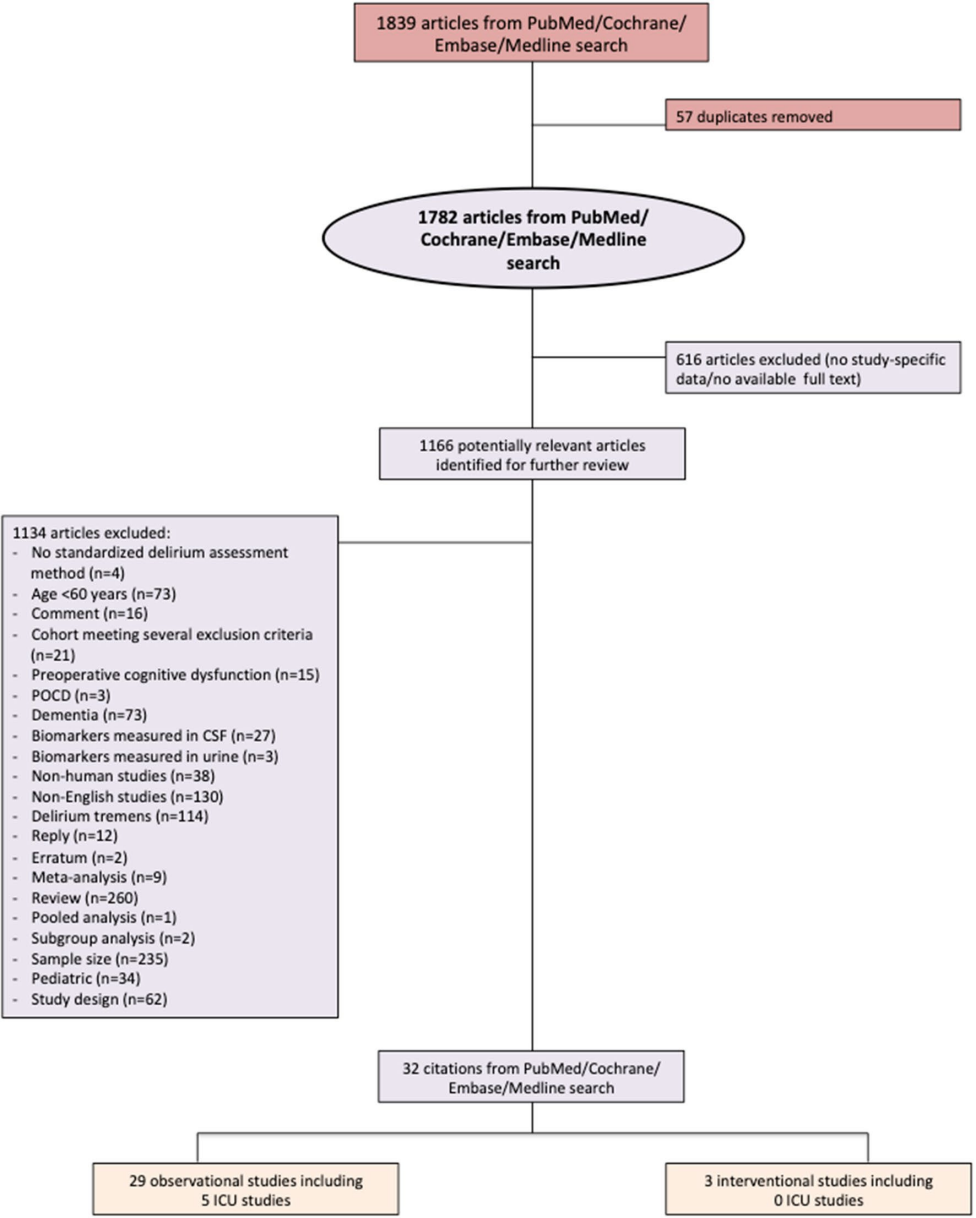
Flow diagram according to literature search

In June 2019, a search of the PubMed, Cochrane, Embase, and MEDLINE databases using the search terms “delirium”, “acute brain dysfunction”, “stroke”, “hemorrhagic stroke”, “ischemic stroke”, “traumatic brain injury”, and “septic encephalopathy” [22] AND “biomarker” (term “biomarker” and each biomarker suggested by the authors, Additional file 1: Table S1) for papers published until June 1, 2019, retrieved 1839 publications (Fig. 1). After removal of 57 duplicates, a critical review of the titles and abstracts was performed, and another 616 articles without study-specific data were excluded. Thirty-two studies (29 observational and three interventional) remained after further exclusion of 1134 articles based on title, abstract, or full text as indicated in Fig. 1. The full texts of these remaining studies (Table 1) were reviewed for data extraction by two independent investigators (AH and KT). Due to age limitation a total of 73 publications on mostly mixed populations (sample size ranging from 10 to 1183) and 34 publications on the pediatric cohort have been excluded from our analysis (Fig. 1).

**Table 1:** Summary of current evidence on biomarkers in elderly delirious patients determined by literature search

Biomarker	Study	Country	Medical specialty	ICU	Sample size	Assessment tool	Event rate (%)	Biomarker value	Main findings
Acetylcholine	Larsen (2010)	USA	S		196^a^	DRS-R98	14.3	L	Anticholinergic treatment (olanzapine) associated with significantly lower incidence of delirium
					204	DSM-III	40.2		
*Adenylate kinase*									
Albumin	**Zhang (2018)**	China	S	Yes	700	CAM-ICU	15.9	L	Preoperative severe hypoalbuminemia (≤ 30 g/L) was associated with increased risk of postoperative delirium
	Guo (2016)	China	S		572	CAM, DSM-IV	21	L	Older age, history of stroke, lower albumin, higher blood glucose, higher total bilirubin, higher CRP, longer surgery duration, and higher volume of red blood cell transfusions are independent risk factors for postoperative delirium
	Capri (2014)	Italy	S		351	CAM	13.4	ND	High preoperative IL-6 level is a risk factor for postoperative delirium
	Larsen (2010)	USA	S		196^a^	DRS-R98	14.3	L	Anticholinergic treatment (olanzapine) associated with significantly lower incidence of delirium
					204	DSM-III	40.2		
	Lee (2010)	Korea	S		81	CAM	13.6	L	Albumin level before surgery significantly lower in patients developing postoperative delirium
Amyloid β1-40	**Sun (2016)**	China	S		257	CAM	21.8	H	Elevated levels of inflammatory cytokines, cortisol, and amyloid β1-40 after surgery under general anesthesia may be involved in the onset of postoperative delirium among elderly oral cancer patients
ASAT	Plaschke (2016)	Germany	M		100	NuDESC	29	L	Plasma ChEA (AChE and BChE) not associated with delirium
	Guo (2016)	China	S		572	CAM, DSM-IV	21	ND	Older age, history of stroke, lower albumin, higher blood glucose, higher total bilirubin, higher CRP, longer surgery duration, and higher volume of red blood cell transfusions are independent risk factors for postoperative delirium
BDNF	**Brum (2015)**	Brazil	M		70	CAM	NA	L	BDNF levels significantly lower in delirium in oncology inpatients
*Cholecystokinin*									
Cholinesterase	**Plaschke (2016)**	Germany	M		100	NuDESC	29	ND	Plasma ChEA (AChE and BChE) not associated with delirium
	**Cerejeira (2012)**	Portugal	S		101	CAM, DSM-IV	36.6	L	Delirium associated with dysfunctional interaction between cholinergic and immune systems
Cortisol	**Sun (2016)**	China	S		257	CAM	21.8	H	Elevated levels of inflammatory cytokines, cortisol, and amyloid β1-40 after surgery under general anesthesia may be involved in the onset of postoperative delirium among elderly oral cancer patients
*Creatine kinase*									
*Creatine kinase BB*									
*CREB*									
CRP	**Slor (2019)**	The Netherlands	S		121	CAM, DRS-R98	33.1	ND^d^	CRP level trajectory after hip surgery coincides with delirium from the second day after surgery
	Miao (2018)	China	S		112	DSM-IV	43.8	H	Potential roles of neopterin in pathophysiology and prediction of delirium in elderly patients after open abdominal surgery
	**Vasunilashorn (2018)**	USA	S		560	CAM	24	NA	The signature of postoperative delirium is dynamic, with some proteins important prior to surgery (risk markers: CRP and AZGP1) and others during delirium (disease markers: IL-2, IL-6, and CRP). CRP, AZGP1, and SERPINA3 were identified as top set of delirium-related proteins
	**Cizginer (2017)**	USA	S		556	CAM	24	ND	Vocabulary knowledge, cognitive activities, and education significantly modified association of CRP and postoperative delirium
	**Vasunilashorn (2017)**	USA	S		560	CAM, Chart Review	24	H	High preoperative and postoperative day 2 CRP are independently associated with incidence of delirium
	Egberts (2017)	The Netherlands	M		86	DSM-IV	15.1	H	No significant difference of CRP level among delirious and non-delirious patients
	Plaschke (2016)	Germany	M		100	NuDESC	29	H	Plasma ChEA (AChE and BChE) is not associated with delirium
	**Nguyen (2016)**	Belgium	M + S	Yes	101	CAM-ICU	78	ND	High prolactin levels possible risk factor for delirium in septic patients
	**Sun (2016)**	China	S		257	CAM	21.8	H	Elevated levels of inflammatory cytokines, cortisol, and amyloid β1-40 after surgery under general anesthesia may be involved in the onset of postoperative delirium among elderly oral cancer patients
	**Ritchie (2014)**	UK	M		710	CAM	12.3	H	Association between elevated CRP and delirium
	Guo (2016)	China	S		572	CAM, DSM-IV	21	H	Older age, history of stroke, lower albumin, higher blood glucose, higher total bilirubin, higher CRP, longer surgery duration, and higher volume of red blood cell transfusions are independent risk factors for postoperative delirium
	Cerejeira (2012)	Portugal	S		101	CAM, DSM-IV	36.6	H	Delirium is associated with unbalanced inflammatory response
	**Lee (2011)**	Korea	S		65	K-DRS-98	28	H	CRP levels within 24 and 72 h after hospitalization are significantly higher in patients with delirium
	**Beloosesky (2004)**	Israel	S		32	CAM	31.3	H	CRP kinetics over 30 days after hip surgery is significantly associated with delirium and cardiovascular complications
*Dopamine*									
*Histamine H1*									
*Heat Shock Protein 70*									
IL-2	**Capri (2014)**	Italy	S		351	CAM	13.4	ND	High preoperative IL-6 level is a risk factor for postoperative delirium
	**Vasunilashorn (2018)**	USA	S		560	CAM	24	NA	The signature of postoperative delirium is dynamic, with some proteins important prior to surgery (risk markers: CRP and AZGP1) and others at the time of delirium (disease markers: IL-2, IL-6, and CRP). CRP, AZGP1, and SERPINA3 were identified as top set of delirium-related proteins
IL-6	Gao (2018)	China	S	Yes	64	CAM-ICU	15.6	NA	TEAS can alleviate POD in older patients with silent lacunar infarction and may be related to reduced neuroinflammation by lowering BBB permeability
	Miao (2018)	China	S		112	DSM-IV	43.8	H	Potential roles of neopterin in pathophysiology and prediction of delirium in elderly patients after open abdominal surgery
	**Vasunilashorn (2018)**	USA	S		560	CAM	24	NA	The signature of postoperative delirium is dynamic, with some proteins important prior to surgery (risk markers: CRP and AZGP1) and others at the time of delirium (disease markers: IL-2, IL-6, and CRP). CRP, AZGP1, and SERPINA3 were identified as top set of delirium-related proteins
	**Kuswardhani (2017)**	Indonesia	M		60	MDAS	NA	NA	CACI score, IL-6 levels, and sepsis have a strong relationship with delirium severity
	**Xin (2017)**	China	S		60^c^	NuDESC	11.7	ND	TNF-α significantly associated with postoperative delirium
					60		38.3		
	**Sun (2016)**	China	S		257	CAM	21.8	H	Elevated levels of inflammatory cytokines, cortisol, and amyloid β1-40 after surgery under general anesthesia may be involved in the onset of postoperative delirium among elderly oral cancer patients
	**Capri (2014)**	Italy	S		351	CAM	13.4	H	High preoperative IL-6 level is a risk factor for postoperative delirium
	**Jia (2014)**	China	S		117^b^	DRS-R98	3.4	H	The lower incidence of delirium is at least partly attributable to the reduced systemic inflammatory response mediated by IL-6
					116		12.9		
	Cerejeira (2012)	Portugal	S		101	CAM, DSM-IV	36.6	H	Delirium is associated with unbalanced inflammatory response
	**van Munster (2011)**	The Netherlands	M + S		870	CAM	35.7	NA	Functional genetic variations in the IL-6, IL-6R, and IL-8 genes are not associated with delirium
	**van Munster (2008)**	The Netherlands	S		98	CAM, DOS, DRS-R98	NA	H	Patients with hyperactive or mixed subtype of delirium had significantly higher IL-6 levels than patients with hypoactive delirium. IL-6 and IL-8 may contribute to pathogenesis of postoperative delirium
IL-8	**Xin (2017)**	China	S		60^c^	NuDESC	11.7	ND	TNF-α significantly associated with postoperative delirium
					60		38.3		
	**Capri (2014)**	Italy	S		351	CAM	13.4	ND	High preoperative IL-6 level is a risk factor for postoperative delirium
	Cerejeira (2012)	Portugal	S		101	CAM, DSM-IV	36.6	H	Delirium is associated with unbalanced inflammatory response
	**van Munster (2011)**	The Netherlands	M + S		870	CAM	35.7	NA	Functional genetic variations in the IL-6, IL-6R, and IL-8 genes are not associated with delirium
	**van Munster (2008)**	The Netherlands	S		98	CAM, DOS, DRS-R98	NA	H	IL-6 and IL-8 may contribute to pathogenesis of postoperative delirium
*IL*-*18*									
*Lactate dehydrogenase*									
Leptin	**Chen (2014)**	China	S		186	CAM	37.6	L	Preoperative plasma leptin level may be a useful, complementary tool to predict delirium in general and prolonged delirium in elderly patients after hip surgery
	**Sanchez (2013)**	Colombia	M + S		115	CAM, DSM-IV	23.5	L	Leptin levels could be a useful clinical biomarker to establish risk in elderly patients
Neopterin	**Egberts (2019)**	The Netherlands	S	Yes	211	CAM-ICU, DSM-IV	38.4	H	Acutely ill medical patients with delirium had higher levels of neopterin and higher phenylalanine/tyrosine ratios after elective cardiac surgery
	**Miao (2018)**	China	S		112	DSM-IV	43.8	H	Potential roles of neopterin in pathophysiology and prediction of delirium in elderly patients after open abdominal surgery
*NSE*									
*PI3K*									
Procalcitonin	**Sun (2016)**	China	S		257	CAM	21.8	H	Elevated levels of inflammatory cytokines, cortisol, and amyloid β1-40 after surgery under general anesthesia may be involved in the onset of postoperative delirium among elderly oral cancer patients
*Protein C*									
S-100β	Gao (2018)	China	S	Yes	64	CAM-ICU	15.6	NA	TEAS can alleviate POD in older patients with silent lacunar infarction and may be related to reduced neuroinflammation by lowering BBB permeability
	**Xin (2017)**	China	S		60^c^	NuDESC	11.7	ND	TNF-α is significantly associated with postoperative delirium
					60		38.3		
*SDNF*									
Thioredoxin	**Wu (2017)**	China	S		192	CAM	36.5	H	Thioredoxin in postoperative serum may be a potential biomarker to predict postoperative delirium and POCD in elderly patients
TNF-α	Gao (2018)	China	S	Yes	64	CAM-ICU	15.6	NA	TEAS can alleviate POD in older patients with silent lacunar infarction and may be related to reduced neuroinflammation by lowering BBB permeability
	**Xin (2017)**	China	S		60^c^	NuDESC	11.7	H	TNF-α is significantly associated with postoperative delirium
					60		38.3		
	Brum (2015)	Brazil	M		70	CAM	NA	ND	No cross-sectional relationship of BDNF and TNF-α blood levels with delirium in oncology inpatients has been demonstrated
	**Capri (2014)**	Italy	S		351	CAM	13.4	ND	High preoperative IL-6 level is a risk factor for postoperative delirium
	Cerejeira (2012)	Portugal	S		101	CAM, DSM-IV	36.6	ND	Delirium is associated with unbalanced inflammatory response (see CRP, IL-6, and IL-8)
8-iso-prostaglandin F2α	**Zheng (2016)**	China	S		182	CAM	37.4	H	Postoperative plasma 8-iso-prostaglandin F2α levels may have the potential to predict postoperative delirium and POCD in elderly patients
*Additional reported biomarkers resulting from literature search*									
AZGP1	**Vasunilashorn (2018)**	USA	S		560	CAM	24	L	The signature of postoperative delirium is dynamic, with some proteins important prior to surgery (risk markers: CRP and AZGP1) and others at the time of delirium (disease markers: IL-2, IL-6, and CRP). CRP, AZGP1, and SERPINA3 were identified as top set of delirium-related proteins
BUN	Miao (2018)	China	S		112	DSM-IV	43.8	ND	Potential roles of neopterin in pathophysiology and prediction of delirium in elderly patients after open abdominal surgery
	**Kuswardhani (2017)**	Indonesia	M		60	MDAS	NA	NA	BUN only has a weak role in delirium severity in elderly patients with infection
Creatinine	Miao (2018)	China	S		112	DSM-IV	43.8	ND	Potential roles of neopterin in pathophysiology and prediction of delirium in elderly patients after open abdominal surgery
	Bakker (2012)	The Netherlands	S	Yes	201	CAM-ICU	31.3	H	Creatinine level is one of the three independent risk factors for delirium after cardiac surgery
ILGF-1	Miao (2018)	China	S		112	DSM-IV	43.8	L	Potential roles of neopterin in pathophysiology and prediction of delirium in elderly patients after open abdominal surgery
IL-1β	**Xin (2017)**	China	S		60^c^	NuDESC	11.7	ND	TNF-α significantly associated with postoperative delirium
					60	NuDESC	38.3	ND	
	**Capri (2014)**	Italy	S		351	CAM	13.4	ND	High preoperative IL-6 level is a risk factor for postoperative delirium
	Cerejeira (2012)	Portugal	S		101	CAM, DSM-IV	36.6	ND	Delirium is associated with unbalanced inflammatory response (see CRP, IL-6, and IL-8)
IL-12	**van Munster (2008)**	The Netherlands	S		98	CAM, DOS, DRS-R98	NA	ND	IL-6 and IL-8 may contribute to the pathogenesis of postoperative delirium
ILGF-1	**Chu (2016)**	China	S		103	CAM, DSM-IV	22.3	ND	No association found between preoperative ILGF-1 levels and postoperative delirium
MMP-9	Gao (2018)	China	S	Yes	64	CAM-ICU	15.6	NA	TEAS can alleviate POD in older patients with silent lacunar infarction and may be related to reduced neuroinflammation by lowering BBB permeability
NLR	**Egberts (2017)**	The Netherlands	M		86	DSM-IV	15.1	H	NLR levels are significantly increased in patients with delirium
Prolactin	**Nguyen (2016)**	Belgium	M + S	Yes	101	CAM-ICU	78	H	High prolactin levels are a possible risk factor for delirium in septic patients
Phenylalanine–tyrosine ratio	**Egberts (2019)**	The Netherlands	S	Yes	211	CAM-ICU, DSM-IV	38.4	H	Acutely ill medical patients with delirium had higher levels of neopterin and higher phenylalanine–tyrosine ratios after elective cardiac surgery
SERPINA3	**Vasunilashorn (2018)**	USA	S		560	CAM	24	H	The signature of postoperative delirium is dynamic, with some proteins important prior to surgery (risk markers: CRP and AZGP1) and others at the time of delirium (disease markers: IL-2, IL-6, and CRP). CRP, AZGP1, and SERPINA3 were identified as top set of delirium-related proteins

Data are presented as event rate of delirium. Biomarker value refers to comparison of biomarker level in delirious patients to non-delirious patients. Authors bold, main biomarker investigated in the indicated study; biomarkers italic = no data available with the search terms used; authors underlined, interventional studies

*M* medical, *S* surgical, *H* biomarker level higher in delirious patients, *L* biomarker level lower in delirious patients, *NA* not applicable, *ND* no difference of biomarker level among groups, *AChE* acetylcholinesterase, *ASAT* aspartate aminotransferase, *AZGP1* alpha-2 glycoprotein, *BBB* blood–brain barrier, *BChE* butyrylcholinesterase, *BDNF* brain-derived neurotrophic factor, *BUN* blood urea nitrogen, *CAM* Confusion Assessment Method, *ChEA* cholinergic enzyme activity, *CREB* cyclic AMP response element-binding protein, *CRP* C-reactive protein, *DOS* Delirium Observation Scale, *DRS*-*R98* Delirium Rating Scale Revised-98, *DSM* Diagnostic and Statistical Manual of Mental Disorders, *ICU* intensive care unit, *IL* interleukin, *ILGF*-*1* insulin-like growth factor-1, *IQR* interquartile range, *K*-*DRS*-*98* Korean version of DRS, *MDAS* Memorial Delirium Assessment Scale, *MMP*-*9* metalloproteinase-9, *NLR* neutrophil–lymphocyte ratio, *NSE* neuron-specific enolase, *NuDESC* Nursing Delirium Screening Scale, *PI3K* phosphatidylinositol-3-kinases, *POCD* postoperative cognitive dysfunction, *SDNF* striatal-derived neuronotrophic factor, *SERPINA3* alpha 1-antichymotrypsin, *TEAS* transcutaneous electrical acupoint stimulation, *TNF* tumor necrosis factor

^a^Experimental arm (olanzapine)

^b^Experimental arm (fast-track surgery)

^c^Experimental arm (hypertonic saline)

^d^On postoperative day 1; significant difference thereafter

### Study characteristics

All 32 studies were published before May 2019 and included information on 7610 patients. Twenty-four studies reported data from surgical patients [23–46], of which two studies analyzed the same patient cohort [29, 30]. Of these 24 studies, 12 reported data collected from delirium high-risk surgical cohorts: Two studies reported data from cardiac surgery [39, 42], and ten reported data from hip surgery patients [23, 24, 28, 32, 33, 35–37, 41, 45]. Five studies reported data from medical patients (1026 patients) [47–51], and three studies reported data from a mixed cohort (i.e., surgical and medical patients or not defined; 1086 patients) [52–54]. Twenty-nine were observational studies, and three were interventional studies (outlined in more detail below).

### Biomarkers

The authors initially screened for biomarkers already known as possible markers of delirium (Additional file 2: Table S2). A second comprehensive screening of the literature was for biomarkers mentioned particularly in the context of other neurological diseases, but bearing a possible association with delirium, including dementia, delirium tremens, hypoxic brain injury, and Parkinson’s disease (Additional file 2: Table S2). These searches resulted in 11 additional biomarkers that were also investigated in the context of delirium in the elderly (Additional file 2: Table S2).

Biomarkers were grouped according to their biochemical function (i.e., cytokine, enzyme, growth factor, hormone, metabolic product, neuronotrophic factor, neurotransmitter, transcription factor, transport protein, or other; Table 2). Overall, 20 biomarkers were reported to detect or to be associated with delirium (Table 3). Of these, higher levels of 14 biomarkers [i.e., IL-6, cortisol, prolactin, amyloid, creatinine, C-reactive protein (CRP), neopterin, metalloproteinase-9 (MMP-9), neutrophil–lymphocyte ratio (NLR), phenylalanine–tyrosine ratio, procalcitonin, thioredoxin, serpin family A member 3 (SERPINA3 (alpha 1-antichymotrypsin)), and 8-iso-prostaglandin F2α] and lower levels of 6 biomarkers [i.e., brain-derived neurotrophic factor (BDNF), leptin, acetylcholine, albumin, insulin-like growth factor-1 (ILGF-1), and alpha-2 glycoprotein (AZGP1)] were reported in delirious patients. However, apart from CRP clinical relevance of the presented biomarkers was either questioned or denied by the authors (Table 3). With the exception of IL-1β and IL-12 all inflammatory biomarkers could be linked to delirium (Additional file 3: Table S3; Additional file 4: Table S4). Moreover, a connection to delirium was found for all four biomarkers of metabolism (Additional file 3: Table S3; Additional file 4: Table S4).

**Table 2:** Grouping of suggested biomarkers of delirium according to their main function

Function	Assigned biomarker	Established clinical use
Cytokine	IL-1β	Inflammatory marker
	IL-2	
	IL-6	
	IL-8	
	IL-12	
	IL-18	
	TNF-α	
Enzyme	Adenylate kinase	Marker of liver cell damage
	ASAT	Marker of cell damage
	Cholinesterase	Marker of liver synthetic function
	CK	Marker of muscle damage
	CK-BB	Tumor marker
	LDH	Marker of tissue breakdown
	NSE	Tumor marker; brain damage marker
	PI3K	
Growth factor	BDNF	Diagnosis of growth hormone deficiency; marker of pituitary function
	IGF-1	
Hormone	Cholecystokinin	
	Cortisol	Marker of adrenal function
	Leptin	
	Prolactin	Marker of pituitary gland/hypothalamus function; fertility assessment; tumor marker
Metabolic product	Amyloid	
	Phenylalanine–tyrosine ratio	
Neuronotrophic factor	S-100β	Tumor marker; brain damage marker
	SDNF	
Neurotransmitter	Acetylcholine	
	Dopamine	
	Histamine	
Transcription factor	CREB	
Transport protein	Albumin	Nutritional marker, negative acute phase protein
Other	AZGP1	
	BUN	Marker of kidney function
	Creatinine	Marker of kidney function
	CRP	Inflammatory marker, positive acute phase protein
	HSP70	
	Metalloproteinase-9	Proinflammatory marker
	Neopterin	Marker of infection
	NLR	
	Procalcitonin	Proinflammatory marker
	Protein C	
	Thioredoxin	
	8-iso-prostaglandin F2α	

*ASAT* aspartate aminotransferase, *AZGP1* alpha-2 glycoprotein, *BDNF* brain-derived neurotrophic factor, *BUN* blood urea nitrogen, *CK* creatine kinase, *CK*-BB creatine kinase BB, *CREB* cyclic AMP response element-binding protein, *CRP* C-reactive protein, *HSP70* heat shock protein 70, *IL* interleukin, *IGF*-*1* insulin-like growth factor-1, *LDH* lactate dehydrogenase, *NLR* neutrophil–lymphocyte ratio, *NSE* neuron-specific enolase, *PI3K* phosphatidylinositol-3-kinases, *PCT* procalcitonin, *SDNF* striatal-derived neuronotrophic factor, *TNF* tumor necrosis factor

**Table 3:** Role of suggested biomarkers of delirium according to literature reports

Function	Assigned biomarker	Biomarker of delirium	Clinically useful
Cytokine	IL-1β	–	–
	IL-2	?	–
	IL-6	+	–
	IL-8	?	–
	IL-12	–	
	IL-18	NR	–
	TNF-α	?	–
Enzyme	Adenylate kinase	NR	–
	ASAT	?	–
	Cholinesterase	?	–
	CK	NR	–
	CK-BB	NR	–
	LDH	NR	–
	NSE	NR	–
	PI3K	NR	–
Growth factor	BDNF	+	–
	ILGF-1	+	–
Hormone	Cholecystokinin	NR	–
	Cortisol	+	–
	Leptin	+	–
	Prolactin	+	–
Metabolic product	Amyloid	+	?
	Phenylalanine–tyrosine ratio	+	?
Neuronotrophic factor	S-100β	–	–
	SDNF	NR	–
Neurotransmitter	Acetylcholine	+	–
	Dopamine	NR	–
	Histamine	NR	–
Transcription factor	CREB	NR	–
Transport protein	Albumin	+	?
Other	AZGP1	+	–
	BUN	?	–
	Creatinine	+	?
	CRP	+	+
	HSP70	NR	–
	Metalloproteinase-9	+	?
	Neopterin	+	?
	NLR	+	?
	Procalcitonin	+	?
	Protein C	NR	–
	Thioredoxin	+	–
	SERPINA3	+	–
	8-iso-prostaglandin F2α	+	–

*NR* nothing reported in the literature, + correlation with delirium, – no correlation with delirium, *ASAT* aspartate aminotransferase, *AZGP1* alpha-2 glycoprotein, *BDNF* brain-derived neurotrophic factor, *BUN* blood urea nitrogen, *CREB* cyclic AMP response element-binding protein, *CRP* C-reactive protein, *IL* interleukin, *ILGF*-*1* insulin-like growth factor-1, *NLR* neutrophil–lymphocyte ratio, *NSE* neuron-specific enolase, *PI3K* phosphatidylinositol-3-kinases, *SDNF* striatal-derived neuronotrophic factor, *SERPINA3* alpha 1-antichymotrypsin, *TNF* tumor necrosis factor

### Surgical cohort

Overall, 19 studies reported on the incidence of delirium. Event rate of postoperative delirium ranged from 13.4 to 43.8% (Table 1). Four studies reported data from ICU patients (delirium incidence range 15.8 to 43.8%; Table 1) [39]. Within the studies reporting data from surgical patients, three were interventional and as such did not group patients into delirious and non-delirious prior to investigation. Each of these three studies showed a lower incidence of delirium in the experimental arm (Table 1): One compared olanzapine to placebo (delirium incidence 14.3% vs. 40.2%) [23], one compared fast-track surgery to the standard procedure (delirium incidence 3.4% vs. 12.9%) [34], and one compared hypertonic to normal saline (delirium incidence 11.7% vs. 38.3%) [33].

#### Biomarker assessment of postoperative delirium

Biomarkers reported from investigations of surgical cohorts are shown in Table 1. Two studies from the surgical population and one from the medical population evaluated the role of the acetylcholine pathway in delirium. Whereas anticholinergic treatment was suggested as a promising strategy to reduce the incidence of delirium [23], reports on cholinesterase were not as clear [28]. Albumin was the main focus of one study [43], but also evaluated in four other studies resulting from our literature search [23–26]. It was almost consistently reported to be lower in delirious patients. Amyloid β1-40, a protein associated with dementia, was the main focus of Sun and colleagues [27] who reported a possible role in the detection of delirium. No difference in ASAT was found among postoperatively delirious and non-delirious patients. Like albumin, ASAT was not the focus of the study cited here [24]. Among inflammatory markers, increased procalcitonin [27], CRP [24, 27, 28, 30–32, 41, 45, 46], and IL-6 [25, 27, 28, 34, 35, 44, 46] were consistently reported to be associated with delirium with the exception of one study that found no increase in IL-6 levels [33]. Of note, in this study, IL-6 was collected early (i.e., venous blood was drawn at 06:00 on the first day after surgery). Cortisol [27] and leptin were the two reported hormones with a possible link to postoperative delirium. Leptin levels were significantly lower in patients with delirium compared to those without [36]. Finally, AZGP1 [29], MMP-9 [44], neopterin [42], phenylalanine–tyrosine ratio [42], SERPINA3 [29], thioredoxin [37], and 8-iso-prostaglandin F2α [38] were linked to postoperative delirium.

### Medical cohort

Within the studies reporting data from medical patients, three studies reported an incidence of delirium ranging from 12.3 to 29% (Table 1).

#### Biomarker assessment of delirium in elderly patients admitted for medical reasons

Biomarkers reported from investigations on patients admitted for medical reasons are outlined in Table 1. The study reporting results on the assessment of cholinesterases (i.e., acetylcholinesterase and butyrylcholinesterase) in medical patients found no association with delirium [47]. As opposed to the surgical cohort, ASAT was found to be lower in delirious patients admitted for medical reasons [47]. Lower BDNF levels were clearly linked to delirium in oncology patients [48]. Findings on CRP [47, 49, 50] and IL-6 [51] concurred with those reported from the surgical cohorts. NLR was additionally linked to delirium [49].

### Mixed cohort

The three studies to report data from both, medical and surgical patients, reported a combined incidence of delirium of 23.5% (Sanchez [54]) and 35.7% (Van Munster [53]) in non-ICU patients, and of 78% (Nguyen [52]) in ICU patients with sepsis (Table 1).

#### Biomarker assessment of delirium in mixed cohorts of elderly patients

Biomarkers reported from investigations of mixed cohorts are also outlined in Table 1. The two markers reported to have a possible association with delirium in mixed patient cohorts were leptin [54] and prolactin (ICU cohort; [52]).

## Discussion

Our updated findings are consistent with a review published 7 years ago, in 2011, by Khan and colleagues [19] who had reported a lack of evidence for the clinical use of biomarkers to aid in earlier detection, prevention, or treatment of delirium. As long as there are no adequate means of intervention, altered levels of biomarkers are unlikely to initiate any change in clinical practice. So far, no biomarker has been identified that would enable development of treatment strategies to lower the incidence, severity, or duration of delirium. A useful biomarker needs to be easily identifiable and reliable to enable targeted therapy while being cost-effective. These criteria were not applicable for any biomarker found in this literature review. Of note, we only reported data from patients aged 60 and older, as opposed to the review by Khan and colleagues [19].

Our main conclusion that none of the biomarkers described help to lower the incidence of delirium is based on several considerations. Pathophysiological explanations such as those addressed by the neurotransmitter hypothesis have already been described nearly 40 years ago [55]. Despite the knowledge of the role of neurotransmitters in the pathophysiology of delirium, with central cholinergic deficiency remaining the leading hypothesis [56], we have not yet been able to find a way to decrease the incidence of delirium. Dopamine excess and inflammation are important assumptions competing with or contributing to the hypothesis of cholinergic deficiency [57], but the focus should lie on modifiable factors causing delirium [18]. This includes stress reduction due to minimization of light and noise disturbances at night and adequate pain management among other things, but also preventive drug therapy in high-risk cohorts [58, 59]. Most importantly, the pathophysiology of delirium remains poorly understood [60] despite being first described by Hippocrates more than 2500 years ago [61]. So on the one hand, our findings are consistent with established theories such as the role of neurotransmitters, inflammation, and stress response in delirium (i.e., reported association of acetylcholine, IL-6, CRP, procalcitonin, and cortisol). On the other hand, there are new approaches that, however, lack sufficient evidence or validation for implementation into everyday clinical practice (i.e., BDNF, leptin, MMP-9, neopterin, phenylalanine–tyrosine ratio, prolactin, NLR, thioredoxin, 8-iso-prostaglandin F2α, AZGP1, and SERPINA3). As such, the latter are not readily available. Delirium biomarkers that are included in standard blood examinations (i.e., albumin and creatinine) may be useful to attribute higher delirium risk but are not targets specific to delirium prevention since they are routinely addressed with respect to other clinical questions. It is nevertheless interesting that an elevated creatinine level was reported to be an independent risk factor for delirium in a cardiac surgery cohort; clinicians should keep this in mind [39].

An almost exclusive association of the numerous investigated inflammatory biomarkers with delirium offers room for and discussion on a novel approach: The implementation of a widely used inflammatory marker (i.e., CRP or procalcitonin) as a diagnostic tool might help facilitate diagnosis of hypoactive delirium. The same can be said for biomarkers of metabolism. Moreover, these biomarkers could be implemented into a tool to assess delirium risk. Finally, the accepted role of amyloid β1-40 for delirium risk has also been confirmed. But clinical relevance here can be reduced to knowledge of its existence, since patients suffering from dementia are known to bear higher risk of delirium [62].

We acknowledge the following limitations of the presented review. First, we did not include any reports on biomarkers with a sample size lower than 10. Important biomarkers to be further explored in larger cohorts may thus have escaped our attention. Second, we focused on patients aged 60 and older, which led to the exclusion of numerous publications reporting other potentially important biomarkers for the elderly population including key articles on delirium biomarkers [63–68]. Interestingly, putative delirium biomarkers such as NSE and S-100β were reported/found to be irrelevant [59]. Third, there is an imbalance between medical and surgical patients investigated. In addition, high-risk surgical groups (i.e., hip and cardiac surgery) are overrepresented, whereas another important high-risk group (i.e., ICU patients) is underrepresented in the reported literature. Last, all observational studies have outlined biomarker levels comparing delirious to non-delirious patients. Overall, delirium is diagnosed clinically by established delirium assessment methods and biomarker groups and could be helpful to diagnose patients where a delirium is not so obvious (i.e., hypoactive delirium). In addition, it is not easy to influence levels of the biomarkers reported here other than by following guidelines (e.g., hygiene directives, pain control, prevention and management of infections, avoidance of unnecessary surgery, and adequate nutrition).

## Conclusion

The concluding observations offer no ground-breaking recommendation for the implementation of a specific biomarker of delirium. Inflammatory biomarkers and biomarkers of metabolism could assist in diagnosing delirium and in assessing delirium risk. Expert opinions state that especially the hypoactive form is frequently undiagnosed even when using established tools to diagnose delirium. The implementation of these biomarkers in delirium assessment tools could represent a new approach. However, authors found inflammatory biomarkers not consistently reported as delirium risk factors. Their level of evidence should first be investigated in a meta-analysis.

## Additional files

**Additional file 1: Table S1.** Terms used for biomarker search in alphabetical order.

**Additional file 2: Table S2.** Overview of biomarkers investigated in this review including references.

**Additional file 3: Table S3** Role of inflammatory and metabolic biomarkers in delirium according to literature reports.

**Additional file 4: Table S4** Sensitivity and specificity analysis of inflammatory biomarkers and biomarkers of metabolism that could assist in diagnosing delirium and in assessing delirium risk.

## Data Availability

Not applicable.

## Appendix: Detailed search strategy

### PubMed

“Delirium” AND “biomarkers”, “delirium” AND “Acetylcholine”, “Delirium” AND “Adenylate kinase”, “Delirium” AND “Myokinase”, “Delirium” AND “Albumin”, “Delirium” AND “Amyloid”, “Delirium” AND “ASAT”, “Delirium” AND “Aspartate transaminase”, “Delirium” AND “Aspartate aminotransferase“, “Delirium” AND “AST”, “Delirium” AND “BDNF”, “Delirium” AND “Brain-derived neurotrophic factor”, “Delirium” AND “Cholezystokinine”, “Delirium” AND “Cholinesterase”, “Delirium” AND “Cortisol”, “Delirium” AND “Creatine kinase”, “Delirium” AND “Creatine phosphokinase”, “Delirium” AND “Creatine kinase BB”, “Delirium” AND “CK-BB”, “Delirium” AND “CREB”, “Delirium” AND “Cyclic AMP response element-binding protein”, “Delirium” AND “CRP”, “Delirium” AND “Dopamine”, “Delirium” AND “Histamine H1”, “Delirium” AND “Heat Shock Protein 70”, “Delirium” AND “IL-2”, “Delirium” AND “Interleukin-2”, “Delirium” AND “IL-6”, “Delirium” AND “Interleukin-6”, “Delirium” AND “IL-8”, “Delirium” AND “Interleukin-8”, “Delirium” AND “IL-18”, “Delirium” AND “Interleukin-18”, “Delirium” AND “Lactate dehydrogenase”, “Delirium” AND “Leptin”, “Delirium” AND “Neopterin”, “Delirium” AND “NSE”, “Delirium” AND “Neuron specific enolase”, “Delirium” AND “Phosphatidylinositol-3-kinases”, “Delirium” AND “Phosphatidylinositol-4,5-bisphosphate 3-kinase”, “Delirium” AND “Phosphatidylinositide 3-kinases”, “Delirium” AND “PI3K”, “Delirium” AND “PCT”, “Delirium” AND “Procalcitonin”, “Delirium” AND “Protein C”, “Delirium” AND “S-100”, “Delirium” AND “S-100beta”, “Delirium” AND “S-100β”, “Delirium” AND “Calcium-binding protein B”, “Delirium” AND “S100 protein”, “Delirium” AND “SDNF”, “Delirium” AND “Mammalian striatal-derived neuronotrophic factor”, “Delirium” AND “Striatal-derived neuronotrophic factor”, “Delirium” AND “Neuronotrophic factor”, “Delirium” AND “Thioredoxin”, “Delirium” AND “TNF-α”, “Delirium” AND “TNF-alpha”, “Delirium” AND “8-iso Prostaglandin F2α”.

no limit [all fields].

### Cochrane

“Delirium” AND “Biomarker”, “Delirium” AND “Acetylcholine”, “Delirium” AND “Adenylate kinase”, “Delirium” AND “Myokinase”, “Delirium” AND “Albumin”, “Delirium” AND “Amyloid”, “Delirium” AND “ASAT”, “Delirium” AND “Aspartate transaminase”, “Delirium” AND “Aspartate aminotransferase”, “Delirium” AND “AST”, “Delirium” AND “BDNF”, “Delirium” AND “Brain-derived neurotrophic factor”, “Delirium” AND “Cholezystokinine”, “Delirium” AND “Cholinesterase”, “Delirium” AND “Cortisol”, “Delirium” AND “Creatine kinase”, “Delirium” AND “Creatine phosphokinase”, “Delirium” AND “Creatine kinase BB”, “Delirium” AND “CK-BB”, “Delirium” AND “CREB”, “Delirium” AND “Cyclic AMP response element-binding protein”, “Delirium” AND “CRP”, “Delirium” AND “Dopamine”, “Delirium” AND “Histamine H1”, “Delirium” AND “Heat Shock Protein 70”, “Delirium” AND “IL-2”, “Delirium” AND “Interleukin-2”, “Delirium” AND “IL-6”, “Delirium” AND “Interleukin-6”, “Delirium” AND “IL-8”, “Delirium” AND “Interleukin-8”, “Delirium” AND “IL-18”, “Delirium” AND “Interleukin-18”, “Delirium” AND “Lactate dehydrogenase”, “Delirium” AND “Leptin”, “Delirium” AND “Neopterin”, “Delirium” AND “NSE”, “Delirium” AND “Neuron specific enolase”, “Delirium” AND “Phosphatidylinositol-3-kinases”, “Delirium” AND “Phosphatidylinositol-4,5-bisphosphate 3-kinase”, “Delirium” AND “Phosphatidylinositide 3-kinases”, “Delirium” AND “PI3K”, “Delirium” AND “PCT”, “Delirium” AND “Procalcitonin”, “Delirium” AND “Protein C”, “Delirium” AND “S-100”, “Delirium” AND “S-100beta”, “Delirium” AND “S-100β”, “Delirium” AND “Calcium-binding protein B”, “Delirium” AND “S100 protein”, “Delirium” AND “SDNF”, “Delirium” AND “Mammalian striatal-derived neuronotrophic factor”, “Delirium” AND “Striatal-derived neuronotrophic factor”, “Delirium” AND “Neuronotrophic factor”, “Delirium” AND “Thioredoxin”, “Delirium” AND “TNF-α”, “Delirium” AND “TNF-alpha”, “Delirium” AND “8-iso Prostaglandin F2α”.

[full text], [human].

Identical search for: “Acute Brain Dysfunction” AND “XXX”; “Stroke” AND “XXX” AND “Delirium”; “Hemorrhagic Stroke” AND “XXX” AND “Delirium”; “Ischemic Stroke” AND “XXX” AND “Delirium”; “Traumatic Brain Injury” AND “XXX” AND “Delirium”; “Septic Encephalopathy” AND “XXX” AND “Delirium”.

### EMBASE/MEDLINE

“Delirium” AND “Biomarker”, “Delirium” AND “Acetylcholine”, “Delirium” AND “Adenylate kinase”, “Delirium” AND “Myokinase”, “Delirium” AND “Albumin”, “Delirium” AND “Amyloid”, “Delirium” AND “ASAT”, “Delirium” AND “Aspartate transaminase”, “Delirium” AND “Aspartate aminotransferase“, “Delirium” AND “AST”, “Delirium” AND “BDNF”, “Delirium” AND “Brain-derived neurotrophic factor”, “Delirium” AND “Cholezystokinine”, “Delirium” AND “Cholinesterase”, “Delirium” AND “Cortisol”, “Delirium” AND “Creatine kinase”, “Delirium” AND “Creatine phosphokinase”, “Delirium” AND “Creatine kinase BB”, “Delirium” AND “CK-BB”, “Delirium” AND “CREB”, “Delirium” AND “Cyclic AMP response element-binding protein”, “Delirium” AND “CRP”, “Delirium” AND “Dopamine”, “Delirium” AND “Histamine H1”, “Delirium” AND “Heat Shock Protein 70”, “Delirium” AND “IL-2”, “Delirium” AND “Interleukin-2”, “Delirium” AND “IL-6”, “Delirium” AND “Interleukin-6”, “Delirium” AND “IL-8”, “Delirium” AND “Interleukin-8”, “Delirium” AND “IL-18”, “Delirium” AND “Interleukin-18”, “Delirium” AND “LDH”, “Delirium” AND “Lactate dehydrogenase”, “Delirium” AND “Leptin”, “Delirium” AND “Neopterin”, “Delirium” AND “NSE”, “Delirium” AND “Neuron specific enolase”, “Delirium” AND “Phosphatidylinositol-3-kinases”, “Delirium” AND “Phosphatidylinositol-4,5-bisphosphate 3-kinase”, “Delirium” AND “Phosphatidylinositide 3-kinases”, “Delirium” AND “PI3K”, “Delirium” AND “PCT”, “Delirium” AND “Procalcitonin”, “Delirium” AND “Protein C”, “Delirium” AND “S-100”, “Delirium” AND “S-100beta”, “Delirium” AND “S-100β”, “Delirium” AND “Calcium-binding protein B”, “Delirium” AND “S100 protein”, “Delirium” AND “SDNF”, “Delirium” AND “Mammalian striatal-derived neuronotrophic factor”, “Delirium” AND “Striatal-derived neuronotrophic factor”, “Delirium” AND “Neuronotrophic factor”, “Delirium” AND “Thioredoxin”, “Delirium” AND “TNF-α”, “Delirium” AND “TNF-alpha”, “Delirium” AND “8-iso Prostaglandin F2α”.

Disease search, “search also as free text in all fields”, “human”, “English”, source “EMBASE” and “Medline”, for publication types “Article”, “Article in press” and “Letter”.

Example: Delirium AND biomarker AND ([article]/lim OR [article in press]/lim OR [letter]/lim) AND [humans]/lim AND [english]/lim AND ([embase]/lim OR [medline]/lim).

## Abbreviations

ASAT: aspartate aminotransferase
AZGP1: alpha-2 glycoprotein
BDNF: brain-derived neurotrophic factor
BUN: blood urea nitrogen
CK: creatine kinase
CK-BB: creatine kinase BB
CAM: Confusion Assessment Method
DRS-R98-T: Delirium Rating Scale Revised-98-T
CREB: cyclic AMP response element-binding protein
CRP: C-reactive protein
DOS: Delirium Observation Screening Scale
DRS: delirium rating scale
DSI: Delirium Symptom Interview
DSM: Diagnostic and Statistical Manual of Mental Disorders
H1: histamine type 1
HSP: heat shock protein
ICB: intracerebral bleeding
ICDSC: Intensive Care Delirium Screening Checklist
ICU: intensive care unit
LDH: lactate dehydrogenase
MDAS: Memorial Delirium Assessment Scale
MoCA: Montreal Cognitive Assessment
NLR: neutrophil–lymphocyte ratio
NSE: neuron-specific enolase
NuDESC: Nursing Delirium Screening Scale
PI3K: phosphatidylinositol-3-kinases
POD: postoperative delirium
SDNF: striatal-derived neuronotrophic factor
SERPINA3: serpin family A member 3 (alpha 1-antichymotrypsin)
TNF: tumor necrosis factor
