# Supplementary material for: Serum biomarkers of delirium in the elderly: a narrative review
Source: Ann Intensive Care. 2019 Jul 1;9:76. doi: 10.1186/s13613-019-0548-1 (PMC6603109; doi:10.1186/s13613-019-0548-1)
Supplement: Supplementary file 1 — Additional file 1: Table S1. Terms used for biomarker search in alphabetical order. [file 13613_2019_548_MOESM1_ESM.docx]

**Additional file 1: Table S1** Terms used for biomarker search in alphabetical order.

| # | Term | Synonyme 1 | Synonyme 2 | Synonyme 3 |
| --- | --- | --- | --- | --- |
| 1 | Acetylcholine |  |  |  |
| 2 | Adenylate kinase | Myokinase |  |  |
| 3 | Albumin |  |  |  |
| 4 | Amyloid |  |  |  |
| 5 | ASAT | [Aspartate transaminase](https://en.wikipedia.org/wiki/Aspartate_transaminase) | Aspartate aminotransferase | AST |
| 6 | BDNF | Brain-derived neurotrophic factor |  |  |
| 7 | Cholezystokinine |  |  |  |
| 8 | Cholinesterase |  |  |  |
| 9 | Cortisol |  |  |  |
| 10 | Creatine kinase | Creatine phosphokinase |  |  |
| 11 | Creatine kinase BB | CK-BB |  |  |
| 12 | CREB | Cyclic AMP response element-binding protein |  |  |
| 13 | CRP |  |  |  |
| 14 | Dopamine |  |  |  |
| 15 | Histamine H1 |  |  |  |
| 16 | Heat Shock Protein 70 |  |  |  |
| 17 | IL-2 | Interleukin-2 |  |  |
| 18 | IL-6 | Interleukin-6 |  |  |
| 19 | IL-8 | Interleukin-8 |  |  |
| 20 | IL-18 | Interleukin-18 |  |  |
| 21 | LDH | Lactate dehydrogenase |  |  |
| 22 | Leptin |  |  |  |
| 23 | Neopterin |  |  |  |
| 24 | NSE | Neuron specific enolase |  |  |
| 25 | Phosphatidylinositol-3-kinases | Phosphatidylinositol-4,5-bisphosphate 3-kinase | Phosphatidylinositide 3-kinases | PI3K |
| 26 | PCT | Procalcitonin |  |  |
| 27 | Protein C |  |  |  |
| 28 | S-100 | S-100beta/β | Calcium-binding protein B | S100 protein |
| 29 | SDNF | Mammalian striatal-derived neuronotrophic factor | Striatal-derived neuronotrophic factor | Neuronotrophic factor |
| 30 | Thioredoxin |  |  |  |
| 31 | TNF-α | TNF-alpha |  |  |
| 32 | 8-iso prostaglandin F2α |  |  |  |

*ASAT* aspartate aminotransferase, *BDNF* brain-derived neurotrophic factor, *CK*-BB creatine kinase BB, *CREB*, cyclic AMP response element-binding protein, *CRP* C-reactive protein, *IL* interleukin, *LDH* lactate dehydrogenase, *NSE* neuron specific enolase, *PI3K* phosphatidylinositol-3-kinases, *PCT* procalcitonin, *SDNF* striatal-derived neuronotrophic factor, *TNF*, tumor necrosis factor
