# Supplementary material for: Serum biomarkers of delirium in the elderly: a narrative review
Source: Ann Intensive Care. 2019 Jul 1;9:76. doi: 10.1186/s13613-019-0548-1 (PMC6603109; doi:10.1186/s13613-019-0548-1)
Supplement: Supplementary file 2 — Additional file 2: Table S2. Overview of biomarkers investigated in this review including references. [file 13613_2019_548_MOESM2_ESM.docx]

**Additional file 2: Table S2** Overview of biomarkers investigated in this review including references

|  | **Reported Biomarkers of Delirium** |
| --- | --- |
|  | Acetylcholine/cholinesterase (1-5) |
|  | Albumin (6, 7) |
|  | Amyloid (8-11) |
|  | Aspartate aminotransferase (ASAT) (12, 13) |
|  | Brain-derived neurotrophic factor (BDNF) (14, 15) |
|  | Cortisol (16-18) |
|  | Creatine kinase (19, 20) |
|  | CRP (21-29) |
|  | Dopamine (5) |
|  | Histamine H1 (30) |
|  | Heat shock protein (HSP) 70 (31) |
|  | Interleukin- (IL)-2 (32) |
|  | IL-6 (17) |
|  | IL-8 (8) |
|  | IL-18 (15, 33) |
|  | Lactate dehydrogenase (LDH) (34) |
|  | Leptin (35) |
|  | Neopterin (36) |
|  | Neuron-specific dnolase (NSE) (14, 15) |
|  | Phosphatidylinositol-3-kinase (PI3K) (37) |
|  | Procalcitonin (21) |
|  | Protein C (29) |
|  | S-100β (1, 14, 18) |
|  | Tumor necrosis factor- (TNF)-α (15, 32) |
|  | Thioredoxin (38) |
|  | 8-iso prostaglandin F2α(39) |
|  | BIOMARKERS WITH POSSIBLE ROLE IN DELIRIUM |
| **Biomarkers reported in Dementia** | Adenylate kinase (40, 41) |
|  | Amyloid |
|  | Cyclic AMP response element-binding protein (CREB) (42, 43) |
| **Biomarkers reported in Delirium Tremens** | Cholecystokinin (44-46) |
| **Biomarkers reported in Hypoxic Brain Injury** | Creatine kinase BB (47) |
|  | S-100β (48) |
| **Biomarkers reported in Hypoxic Brain Injury Parkinson’s Disease** | Striatal-derived neuronotrophic factor (SDNF) (49) |
|  | BIOMARKERS OF DELIRIUM RESULTING FROM LITERATURE SEARCH |
|  | Alpha-2 glycoprotein (AZGP1) (50) |
|  | Blood urea nitrogen (BUN) (51, 52) |
|  | Creatinine (52, 53) |
|  | IL-1β (2, 54, 55) |
|  | IL-12 (56) |
|  | ILGF-1, (52, 57) |
|  | Metalloproteinase-9 (58) |
|  | Neutrophil-lymphocyte ratio (NLR) (59) |
|  | Phenylalanine/tyrosine ratio (60) |
|  | Prolactin (61) |
|  | Alpha-1 antichymotrypsin (SERPINA3) (50) |

**Table references**

1. O'Neal JB, Shaw AD. Predicting, preventing, and identifying delirium after cardiac surgery. Perioper Med (Lond). 2016;5:7.

2. Cerejeira J, Nogueira V, Luis P, Vaz-Serra A, Mukaetova-Ladinska EB. The cholinergic system and inflammation: common pathways in delirium pathophysiology. J Am Geriatr Soc. 2012;60(4):669-75.

3. Trzepacz PT. Anticholinergic Model For Delirium. Semin Clin Neuropsychiatry. 1996;1(4):294-303.

4. John M, Ely EW, Halfkann D, Schoen J, Sedemund-Adib B, Klotz S, et al. Acetylcholinesterase and butyrylcholinesterase in cardiosurgical patients with postoperative delirium. J Intensive Care. 2017;5:29.

5. Trzepacz PT. Is there a final common neural pathway in delirium? Focus on acetylcholine and dopamine. Semin Clin Neuropsychiatry. 2000;5(2):132-48.

6. Kim H, Chung S, Joo YH, Lee JS. The major risk factors for delirium in a clinical setting. Neuropsychiatr Dis Treat. 2016;12:1787-93.

7. Oh ES, Li M, Fafowora TM, Inouye SK, Chen CH, Rosman LM, et al. Preoperative risk factors for postoperative delirium following hip fracture repair: a systematic review. Int J Geriatr Psychiatry. 2015;30(9):900-10.

8. Androsova G, Krause R, Winterer G, Schneider R. Biomarkers of postoperative delirium and cognitive dysfunction. Front Aging Neurosci. 2015;7:112.

9. Adamis D, Treloar A, Martin FC, Gregson N, Hamilton G, Macdonald AJ. APOE and cytokines as biological markers for recovery of prevalent delirium in elderly medical inpatients. Int J Geriatr Psychiatry. 2007;22(7):688-94.

10. Leung JM, Sands LP, Wang Y, Poon A, Kwok PY, Kane JP, et al. Apolipoprotein E e4 allele increases the risk of early postoperative delirium in older patients undergoing noncardiac surgery. Anesthesiology. 2007;107(3):406-11.

11. van Munster BC, Korevaar JC, Zwinderman AH, Leeflang MM, de Rooij SE. The association between delirium and the apolipoprotein E epsilon 4 allele: new study results and a meta-analysis. Am J Geriatr Psychiatry. 2009;17(10):856-62.

12. Findley JK, Park LT, Siefert CJ, Chiou GJ, Lancaster RT, Demoya M, et al. Two routine blood tests-mean corpuscular volume and aspartate aminotransferase-as predictors of delirium tremens in trauma patients. J Trauma. 2010;69(1):199-201.

13. Aldemir M, Ozen S, Kara IH, Sir A, Bac B. Predisposing factors for delirium in the surgical intensive care unit. Crit Care. 2001;5(5):265-70.

14. Grandi C, Tomasi CD, Fernandes K, Stertz L, Kapczinski F, Quevedo J, et al. Brain-derived neurotrophic factor and neuron-specific enolase, but not S100beta, levels are associated to the occurrence of delirium in intensive care unit patients. J Crit Care. 2011;26(2):133-7.

15. Kozak HH, Uguz F, Kilinc I, Uca AU, Serhat Tokgoz O, Akpinar Z, et al. Delirium in patients with acute ischemic stroke admitted to the non-intensive stroke unit: Incidence and association between clinical features and inflammatory markers. Neurol Neurochir Pol. 2017;51(1):38-44.

16. Mu DL, Wang DX, Li LH, Shan GJ, Li J, Yu QJ, et al. High serum cortisol level is associated with increased risk of delirium after coronary artery bypass graft surgery: a prospective cohort study. Crit Care. 2010;14(6):R238.

17. Plaschke K, Fichtenkamm P, Schramm C, Hauth S, Martin E, Verch M, et al. Early postoperative delirium after open-heart cardiac surgery is associated with decreased bispectral EEG and increased cortisol and interleukin-6. Intensive Care Med. 2010;36(12):2081-9.

18. van Munster BC, Bisschop PH, Zwinderman AH, Korevaar JC, Endert E, Wiersinga WJ, et al. Cortisol, interleukins and S100B in delirium in the elderly. Brain Cogn. 2010;74(1):18-23.

19. Anshu Gupta CG, Sarabjeet Khurana. Evaluation of total creatine kinase levels

in a spectrum of neuro-psychiatric disorders in a tertiary neurosciences centre. International Journal of Medicine and Public Health. 2015;5(4).

20. Yazici AB, Yazici E, Erol A. Delirium and High Creatine Kinase and Myoglobin Levels Related to Synthetic Cannabinoid Withdrawal. Case Rep Med. 2017;2017:3894749.

21. McGrane S, Girard TD, Thompson JL, Shintani AK, Woodworth A, Ely EW, et al. Procalcitonin and C-reactive protein levels at admission as predictors of duration of acute brain dysfunction in critically ill patients. Critical Care. 2011;15:R78.

22. Beloosesky Y, Grinblat J, Pirotsky A, Weiss A, Hendel D. Different C-reactive protein kinetics in post-operative hip-fractured geriatric patients with and without complications. Gerontology. 2004;50(4):216-22.

23. Macdonald A, Adamis D, Treloar A, Martin F. C-reactive protein levels predict the incidence of delirium and recovery from it. Age Ageing. 2007;36(2):222-5.

24. Burkhart CS, Dell-Kuster S, Gamberini M, Moeckli A, Grapow M, Filipovic M, et al. Modifiable and nonmodifiable risk factors for postoperative delirium after cardiac surgery with cardiopulmonary bypass. J Cardiothorac Vasc Anesth. 2010;24(4):555-9.

25. Pol RA, van Leeuwen BL, Izaks GJ, Reijnen MM, Visser L, Tielliu IF, et al. C-reactive protein predicts postoperative delirium following vascular surgery. Ann Vasc Surg. 2014;28(8):1923-30.

26. Ritchie CW, Newman TH, Leurent B, Sampson EL. The association between C-reactive protein and delirium in 710 acute elderly hospital admissions. Int Psychogeriatr. 2014;26(5):717-24.

27. Zhang Z, Pan L, Deng H, Ni H, Xu X. Prediction of delirium in critically ill patients with elevated C-reactive protein. J Crit Care. 2014;29(1):88-92.

28. Lemstra AW, Kalisvaart KJ, Vreeswijk R, van Gool WA, Eikelenboom P. Pre-operative inflammatory markers and the risk of postoperative delirium in elderly patients. Int J Geriatr Psychiatry. 2008;23(9):943-8.

29. Girard TD, Ware LB, Bernard GR, Pandharipande PP, Thompson JL, Shintani AK, et al. Associations of markers of inflammation and coagulation with delirium during critical illness. Intensive Care Med. 2012;38(12):1965-73.

30. Clegg A, Young JB. Which medications to avoid in people at risk of delirium: a systematic review. Age Ageing. 2011;40(1):23-9.

31. Mash DC, Duque L, Pablo J, Qin Y, Adi N, Hearn WL, et al. Brain biomarkers for identifying excited delirium as a cause of sudden death. Forensic Sci Int. 2009;190(1-3):e13-9.

32. Kazmierski J, Banys A, Latek J, Bourke J, Jaszewski R. Raised IL-2 and TNF-alpha concentrations are associated with postoperative delirium in patients undergoing coronary-artery bypass graft surgery. Int Psychogeriatr. 2014;26(5):845-55.

33. van den Boogaard M, Kox M, Quinn KL, van Achterberg T, van der Hoeven JG, Schoonhoven L, et al. Biomarkers associated with delirium in critically ill patients and their relation with long-term subjective cognitive dysfunction; indications for different pathways governing delirium in inflamed and noninflamed patients. Crit Care. 2011;15(6):R297.

34. Jang S, Jung KI, Yoo WK, Jung MH, Ohn SH. Risk Factors for Delirium During Acute and Subacute Stages of Various Disorders in Patients Admitted to Rehabilitation Units. Ann Rehabil Med. 2016;40(6):1082-91.

35. Chen XW, Shi JW, Yang PS, Wu ZQ. Preoperative plasma leptin levels predict delirium in elderly patients after hip fracture surgery. Peptides. 2014;57:31-5.

36. Hall RJ, Watne LO, Idland AV, Raeder J, Frihagen F, MacLullich AM, et al. Cerebrospinal fluid levels of neopterin are elevated in delirium after hip fracture. J Neuroinflammation. 2016;13(1):170.

37. Qiu Y, Huang X, Huang L, Tang L, Jiang J, Chen L, et al. 5-HT(1A) receptor antagonist improves behavior performance of delirium rats through inhibiting PI3K/Akt/mTOR activation-induced NLRP3 activity. IUBMB Life. 2016;68(4):311-9.

38. Wu XM, Xu WC, Yu YJ, Han L, Zhang J, Yang LJ. Postoperative serum thioredoxin concentrations correlate with delirium and cognitive dysfunction after hip fracture surgery in elderly patients. Clin Chim Acta. 2017;466:93-7.

39. Zheng YB, Ruan GM, Fu JX, Su ZL, Cheng P, Lu JZ. Postoperative plasma 8-iso-prostaglandin F2alpha levels are associated with delirium and cognitive dysfunction in elderly patients after hip fracture surgery. Clin Chim Acta. 2016;455:149-53.

40. Ahlberg J, Blomstrand C, Ronquist G, Wikkelso C. Dementia--and adenylate kinase activity in cerebrospinal fluid. Acta Neurol Scand. 1985;72(5):525-7.

41. Ansoleaga B, Jove M, Schluter A, Garcia-Esparcia P, Moreno J, Pujol A, et al. Deregulation of purine metabolism in Alzheimer's disease. Neurobiol Aging. 2015;36(1):68-80.

42. Yamamoto-Sasaki M, Ozawa H, Saito T, Rosler M, Riederer P. Impaired phosphorylation of cyclic AMP response element binding protein in the hippocampus of dementia of the Alzheimer type. Brain Res. 1999;824(2):300-3.

43. Han XR, Wen X, Wang YJ, Wang S, Shen M, Zhang ZF, et al. Effects of CREB1 gene silencing on cognitive dysfunction by mediating PKA-CREB signaling pathway in mice with vascular dementia. Mol Med. 2018;24(1):18.

44. Okubo T, Harada S, Higuchi S, Matsushita S. Genetic association between alcohol withdrawal symptoms and polymorphism of CCK gene promoter. Alcohol Clin Exp Res. 1999;23(4 Suppl):11S-2S.

45. Okubo T, Harada S, Higuchi S, Matsushita S. Investigation of quantitative trait loci in the CCKAR gene with susceptibility to alcoholism. Alcohol Clin Exp Res. 2002;26(8 Suppl):2S-5S.

46. Okubo T, Harada S. Polymorphisms of the CCK, CCKAR and CCKBR genes: an association with alcoholism study. J Stud Alcohol. 2001;62(4):413-21.

47. Karkela J, Bock E, Kaukinen S. CSF and serum brain-specific creatine kinase isoenzyme (CK-BB), neuron-specific enolase (NSE) and neural cell adhesion molecule (NCAM) as prognostic markers for hypoxic brain injury after cardiac arrest in man. J Neurol Sci. 1993;116(1):100-9.

48. Bloomfield SM, McKinney J, Smith L, Brisman J. Reliability of S100B in predicting severity of central nervous system injury. Neurocrit Care. 2007;6(2):121-38.

49. Carvey PM, McRae A, Lint TF, Ptak LR, Lo ES, Goetz CG, et al. The potential use of a dopamine neuron antibody and a striatal-derived neurotrophic factor as diagnostic markers in Parkinson's disease. Neurology. 1991;41(5 Suppl 2):53-8; discussion 9-60.

50. Vasunilashorn SM, Ngo LH, Chan NY, Zhou W, Dillon ST, Otu HH, et al. Development of a Dynamic Multi-Protein Signature of Postoperative Delirium. J Gerontol A Biol Sci Med Sci. 2018.

51. Kuswardhani RAT, Sugi YS. Factors Related to the Severity of Delirium in the Elderly Patients With Infection. Gerontol Geriatr Med. 2017;3:2333721417739188.

52. Miao S, Shen P, Zhang Q, Wang H, Shen J, Wang G, et al. Neopterin and mini-mental state examination scores, two independent risk factors for postoperative delirium in elderly patients with open abdominal surgery. J Cancer Res Ther. 2018;14(6):1234-8.

53. Bakker RC, Osse RJ, Tulen JH, Kappetein AP, Bogers AJ. Preoperative and operative predictors of delirium after cardiac surgery in elderly patients. Eur J Cardiothorac Surg. 2012;41(3):544-9.

54. Xin X, Xin F, Chen X, Zhang Q, Li Y, Huo S, et al. Hypertonic saline for prevention of delirium in geriatric patients who underwent hip surgery. J Neuroinflammation. 2017;14(1):221.

55. Capri M, Yani SL, Chattat R, Fortuna D, Bucci L, Lanzarini C, et al. Pre-Operative, High-IL-6 Blood Level is a Risk Factor of Post-Operative Delirium Onset in Old Patients. Front Endocrinol (Lausanne). 2014;5:173.

56. van Munster BC, Korevaar JC, Zwinderman AH, Levi M, Wiersinga WJ, De Rooij SE. Time-course of cytokines during delirium in elderly patients with hip fractures. J Am Geriatr Soc. 2008;56(9):1704-9.

57. Chu CS, Liang CK, Chou MY, Lin YT, Hsu CJ, Chu CL, et al. Lack of Association between Pre-Operative Insulin-Like Growth Factor-1 and the Risk of Post-Operative Delirium in Elderly Chinese Patients. Psychiatry Investig. 2016;13(3):327-32.

58. Gao F, Zhang Q, Li Y, Tai Y, Xin X, Wang X, et al. Transcutaneous electrical acupoint stimulation for prevention of postoperative delirium in geriatric patients with silent lacunar infarction: a preliminary study. Clin Interv Aging. 2018;13:2127-34.

59. Egberts A, Mattace-Raso FU. Increased neutrophil-lymphocyte ratio in delirium: a pilot study. Clin Interv Aging. 2017;12:1115-21.

60. Egberts A, Osse RJ, Fekkes D, Tulen JHM, van der Cammen TJM, Mattace-Raso FUS. Differences in potential biomarkers of delirium between acutely ill medical and elective cardiac surgery patients. Clin Interv Aging. 2019;14:271-81.

61. Nguyen DN, Huyghens L, Schiettecatte J, Smitz J, Vincent JL. High prolactin levels are associated with more delirium in septic patients. J Crit Care. 2016;33:56-61.
