# Supplementary material for: Serum biomarkers of delirium in the elderly: a narrative review
Source: Ann Intensive Care. 2019 Jul 1;9:76. doi: 10.1186/s13613-019-0548-1 (PMC6603109; doi:10.1186/s13613-019-0548-1)
Supplement: Supplementary file 3 — Additional file 3: Table S3 Role of inflammatory and metabolic biomarkers in delirium according to literature reports. [file 13613_2019_548_MOESM3_ESM.docx]

**Additional file 3: Table S3** Role of inflammatory and metabolic biomarkers in delirium according to literature reports

| **Function** | **Assigned biomarker** | **Biomarker of delirum** | **Clinically useful** |
| --- | --- | --- | --- |
| Proinflammatory markers | IL-1β | - | - |
|  | IL-2 | ? | - |
|  | IL-6 | + | - |
|  | IL-8 | ? | - |
|  | IL-12 | - |  |
|  | IL-18 | NR | - |
|  | TNFα | ? | - |
|  | Cortisol | + | - |
|  | Prolactin | + | - |
|  | CRP | + | + |
|  | HSP70 | NR | - |
|  | MMP-9 | + | ? |
|  | Neopterin | + | ? |
|  | NLR | + | ? |
|  | Procalcitonin | + | ? |
|  | SERPINA3 | + | - |
|  | 8-iso prostaglandin F2α | + | - |
| Acute phase proteins | Amyloid | + | ? |
|  | Albumin | + | ? |
|  | CRP | + | + |
|  | Procalcitonin | + | ? |
|  | SERPINA3 | + | - |
| Biomarkers of metabolism | BDNF | + | - |
|  | ILGF-1 | + | ? |
|  | Leptin | + | - |
|  | Phenylalanine/tyrosin ratio | + | ? |

*NR* not reported in the literature, *BDNF* brain-derived neutrotrophic factor, *CRP* C-reactive protein, *HSP70* heat shock protein 70, *IL* interleukin, *ILGF-1* insulin-like growth factor-1, *MMP-9* metalloproteinase-9, *NLR* neutrophil-lymphocyte ratio, *SERPINA3* alpha-1 antichymotrypsin, *TNF* tumor necrosis factor
