# Supplementary material for: Serum biomarkers of delirium in the elderly: a narrative review
Source: Ann Intensive Care. 2019 Jul 1;9:76. doi: 10.1186/s13613-019-0548-1 (PMC6603109; doi:10.1186/s13613-019-0548-1)
Supplement: Supplementary file 4 — Additional file 4: Table S4 Sensitivity and specificity analysis of inflammatory biomarkers and biomarkers of metabolism that could assist in diagnosing delirium and in assessing delirium risk. [file 13613_2019_548_MOESM4_ESM.docx]

**Additional file 4: Table 4** – Sensitivity and specificity analysis of inflammatory biomarkers and biomarkers of metabolism that could assist in diagnosing delirium and in assessing delirium risk.

|  | Time-point of measurement | Patient cohort | Cut-off  value | Sensitivity (%) | Specificity (%) |
| --- | --- | --- | --- | --- | --- |
| **Biomarkers of inflammation** | | | | | |
| **IL-6** | Morning of the day before surgery | Elective gastrointestinal tumor resection via laparotomy (Miao et al., 2018) | 33.5pg/mL | 63 | 98 |
|  |  | Prevalence of orthopedic, vascular and gastrointestinal surgery (Vasunilashorn et al., 2018) | NA | NA | NA |
|  |  | Elderly patients with infection (Kuswardhani et al., 2017) | NA | NA | NA |
|  | 12 hours after surgery | Major non-cardiac surgery (Sun et al., 2016) | 67pg/L | 97 | 98 |
|  | Prior surgery (morning of hospital admission) | Any kind of elective and emergency surgery (Capri et al., 2014) | 7.1pg/mL | 34 | 98 |
|  | First day after surgery | Surgery for colorectal carcinoma (Jia et al., 2014) | 49ng/mL | 100 | 98 |
|  | Postoperative (morning after surgery) | Elective total hip arthroplasty (Cerejeira et al., 2012) | 291pg/mL | 12 | 98 |
|  | During delirium episode and matched with control group | Surgery for hip fractures (van Munster et al., 2008) | 124pg/mL | 11 | 98 |
| **Cortisol** | 12 hours after surgery | Major non-cardiac surgery (Sun et al., 2016) | 606mmol/mL | 79 | 98 |
| **Prolac-tin** | 6-12 hours after ICU admission | Septic patients (Ngyuen et al., 2016) | 450pmol/L | 63** | 64** |
| **CRP** | Morning of the day before surgery | Elective gastrointestinal tumor resection via laparotomy (Miao et al., 2018) | 23.8mg/L*** | 67 | 98 |
|  | Postoperative (no time-point indicated) | Prevalence of orthopedic, vascular and gastrointestinal surgery (Vasunilashorn et al., 2018) | 271ng/mL* | 10 | 98 |
|  | Postoperative (second morning after surgery) | Major elective surgery (Cizginer et al., 2017) | NA | NA | NA |
|  | Postoperative day 2 | Major noncardiac surgery (Vasunilashorn et al., 2017) | NA | NA | NA |
|  | Within 24 hours after admission | Acutely ill patients (Egberts et al., 2017) | 27.9mg/L | 49 | 98 |
|  | Not indicated | Palliative care in cancer (Plaschke et al., 2016) | 254mg/L | 0.7 | 98 |
|  | 12 hours after surgery | Major non-cardiac surgery (Sun et al., 2016) | 99ng/mL | 50 | 98 |
|  | On admission | Acute hospital admissions (Ritchie et al., 2014) | NA | NA | NA |
|  | Prior surgery | Total hip arthroplasty (Guo et al., 2016) | 6.07mg/L | 42 | 98 |
|  | Postoperative (morning after surgery) | Elective total hip arthroplasty (Cerejeira et al., 2012) | 46ng/mL | 0 | 98 |
|  | Postoperative day 1 | Hip surgery (Lee et al., 2011) | 15.62mg/dL | 73 | 98 |
|  | Prior surgery, 48-60h / 7 days / 30 days after surgery | Hip surgery (Beloosesky et al., 2004) | NA | NA | NA |
| **NLR** | Within 24 hours after admission | Acutely ill patients (Egberts et al., 2017) | 7.98 | 57 | 98 |
| **Procal-citonin** | 12 hours after surgery | Major non-cardiac surgery (Sun et al. 2016) | 0.32ng/mL | 20 | 98 |
| **Neopte-rin** | Morning of the day before surgery | Elective gastrointestinal tumor resection via laparotomy (Miao et al., 2018) | 72.8nmol/L | 54 | 98 |
| **SERPI-NA3** | Postoperative day 2 | Prevalence of orthopedic, vascular and gastrointestinal surgery (Vasunilashorn et al., 2018) | 879ng/mL | 0 | 98 |
| **8-iso- prosta-glandin F2α** | Postoperative (morning after surgery) | Surgery for femoral neck fracture or intertrochanteric fracture (Zheng et al., 2016) | 182.4pg/mL | 84 | 78 |
| **Biomarkers of metabolism** | | | | | |
| **BDNF** | Immediately after clinical and psychiatric assessment | Oncology patients (Blum et al., 2015) | 12pg/mL | 34 | 77 |
| **ILGF-1** | Morning of the day before surgery | Elective gastrointestinal tumor resection via laparotomy (Miao et al., 2018) | 13.5nmol/L | 100 | 98 |
| **Leptin** | Before administration of anesthetic agent (experimental) or at study entry (control) | Hip surgery patients (Chen et al., 2014) | 2ng/mL | 72 | 92 |
|  | Morning after admission | Inpatients (Sanchez et al., 2013) | 2ng/mL | 78 | 73 |

Cut-offs have been chosen according to the mean value among the control group (no delirium) indicated in the individual manuscript (i.e., mean + 2x SD = specificity of 98%); otherwise values were directly taken from the manuscript; when median [IQR] was indicated SD was calculated by deducting the 25^th^ percentile from the 75^th^ percentile. NA, sensitivity and specificity cannot be calculated from the information provided in the manuscript.

*age cut-off 65 years

**after surgery in non-delirious controls, calculated by mean + 2xSD

***preoperative cut-off
